# Subtotal diaphyseal replacement of the femur with modular mega-endoprosthesis following interprosthetic fracture. A case report

**DOI:** 10.3205/iprs000183

**Published:** 2024-02-09

**Authors:** Mohamed Ghanem, Christina Pempe, Andreas Roth

**Affiliations:** 1 Department of Orthopedics, Traumatology and Plastic Surgery, University Hospital Leipzig, Germany; 2 Department of Physical Therapy and Rehabilitation, University Hospital Leipzig, Germany

## Abstract

Mega-endoprostheses enable wide management options in the treatment of primary and periprosthetic fracture of the lower extremities. In this study, we report on the use of custom-made subtotal diaphyseal endoprosthetic replacement in treatment of interprosthetic femoral fracture. This procedure is off-label, but in this particular case, it was the safest and most stress-stable treatment option. The implant was delivered within three weeks. The surgical procedure for subtotal replacement of the femoral diaphysis was performed without any intra- or postoperative complication. The duration for the surgical intervention was one hour and 40 minutes. The patient was then mobilized with full weight bearing. At one-year follow-up, the patient did not complain of any pain. The Harris Hip Score HHS improved from 26 to 83 at one-year follow-up, the Western Ontario and McMaster Universities Osteoarthritis Index WOMAC improved from 88 to 16. Mega-endoprostheses enable a wide range of management options in the treatment of primary, peri- and interprosthetic fractures of the lower extremities. In many cases, an individual therapeutic plan is necessary ranging up to the use of custom-made implants.

## Abstract (de)

Megaendoprothesen ermöglichen vielfältige Behandlungsmöglichkeiten bei der Behandlung primärer und periprothetischer Frakturen der unteren Extremitäten. In dieser Studie berichten wir über den Einsatz eines individuell angefertigten subtotalen diaphysären Endoprothesenersatzes bei der Behandlung einer interprothetischen Femurfraktur. Dieses Verfahren ist Off-Label, in diesem speziellen Fall war es aber die sicherste und belastungsstabilste Behandlungsoption. Das Implantat wurde innerhalb von drei Wochen angefertigt. Der chirurgische Eingriff zum subtotalen Ersatz der Femurdiaphyse verlief ohne intra- oder postoperative Komplikationen. Die Dauer des chirurgischen Eingriffs betrug eine Stunde und 40 Minuten. Anschließend wurde die Patientin mit Vollbelastung des operierten Beines mobilisiert. Bei der Nachuntersuchung nach einem Jahr gab die Patientin keinerlei Schmerzen an. Der Harris Hip Score HHS verbesserte sich nach einem Jahr von 26 auf 83, der Western Ontario and McMaster Universities Osteoarthritis Index WOMAC verbesserte sich von 88 auf 16. Mega-Endoprothesen ermöglichen ein breites Spektrum von Managementoptionen bei der Behandlung primärer, periprothetischer und interprothetischer Frakturen der unteren Extremitäten. In vielen Fällen ist eine individuelle Therapieplanung bis hin zum Einsatz individuell angefertigter Implantate erforderlich.

## Introduction

Mega-endoprostheses enable wide management options in the treatment of primary and periprosthetic fracture of the lower extremities [1]. In this study, we report on the use of custom-made subtotal diaphyseal endoprosthetic replacement in treatment of interprosthetic femoral fracture. The aim of the study is to evaluate the short-term outcome of this method. 

## Methods

**Figure 1:**
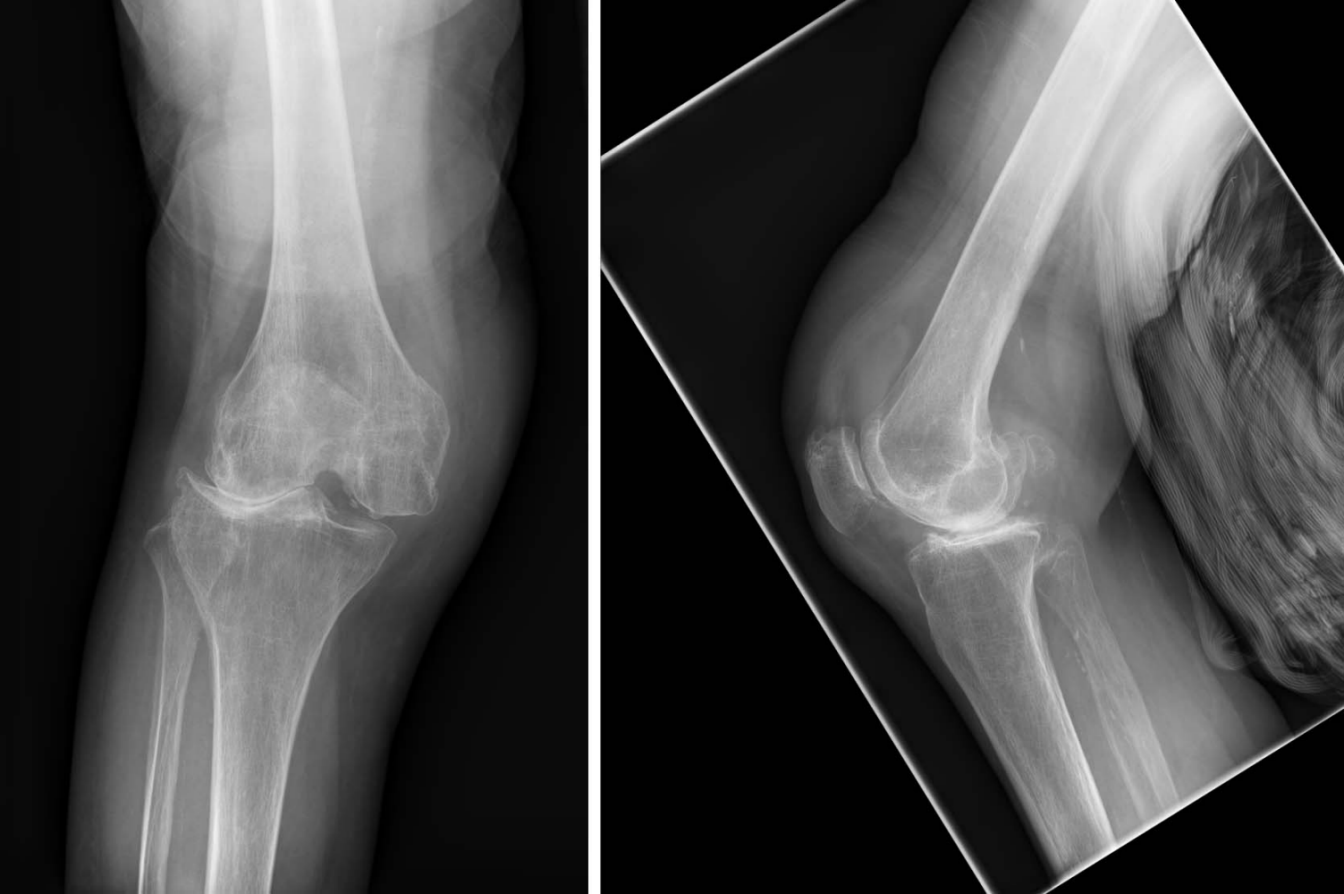
Preoperative X-ray of the right knee joint Fracture of the intercondylar fossa of the distal femur. No fracture of the tibia. No fracture of the fibula. Severe gonarthrosis. Laterally, the joint space is narrowed with pronounced subchondral marginal sclerosis and osteophytes. Subluxation position.

**Figure 2:**
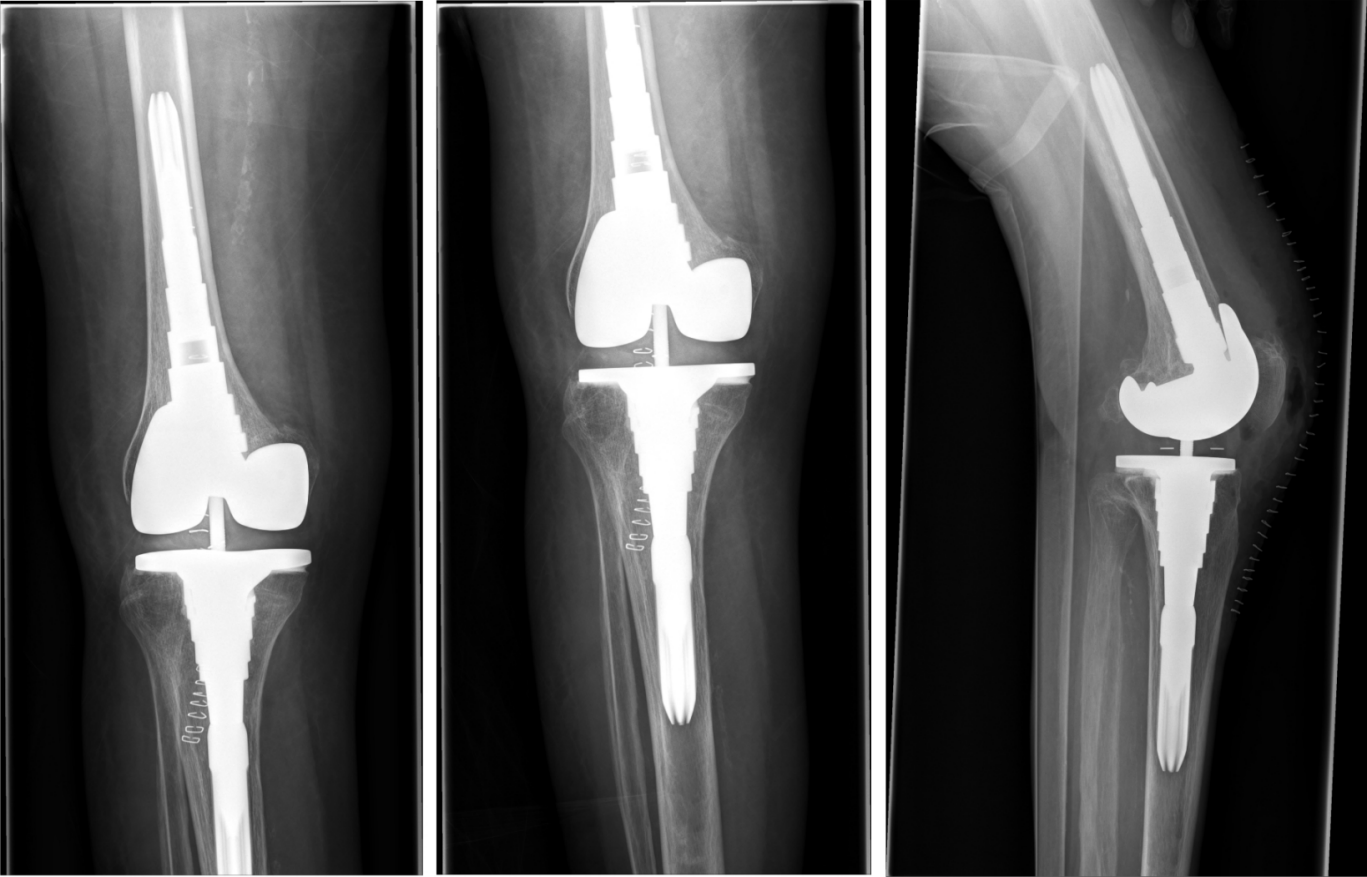
Postoperative x-ray of the right knee joint showing the correct position of the semi-constrained total knee replacement

This is a report on a female patient aged 84 years at time of surgery. The patient was admitted to our center due to right-sided gonarthrosis with partial instability of the collateral ligaments and compression fracture of the medial femoral condyle (Figure 1 (Fig. 1)). In addition, she had rheumatoide polyarthritis. Therefore, we performed total knee replacement (Figure 2 (Fig. 2)) using a valgus-varus constrained LCS-Complete™ Revision System (DePuy Synthes, 325 Paramount Drive, Raynham, MA, USA).

**Figure 3:**
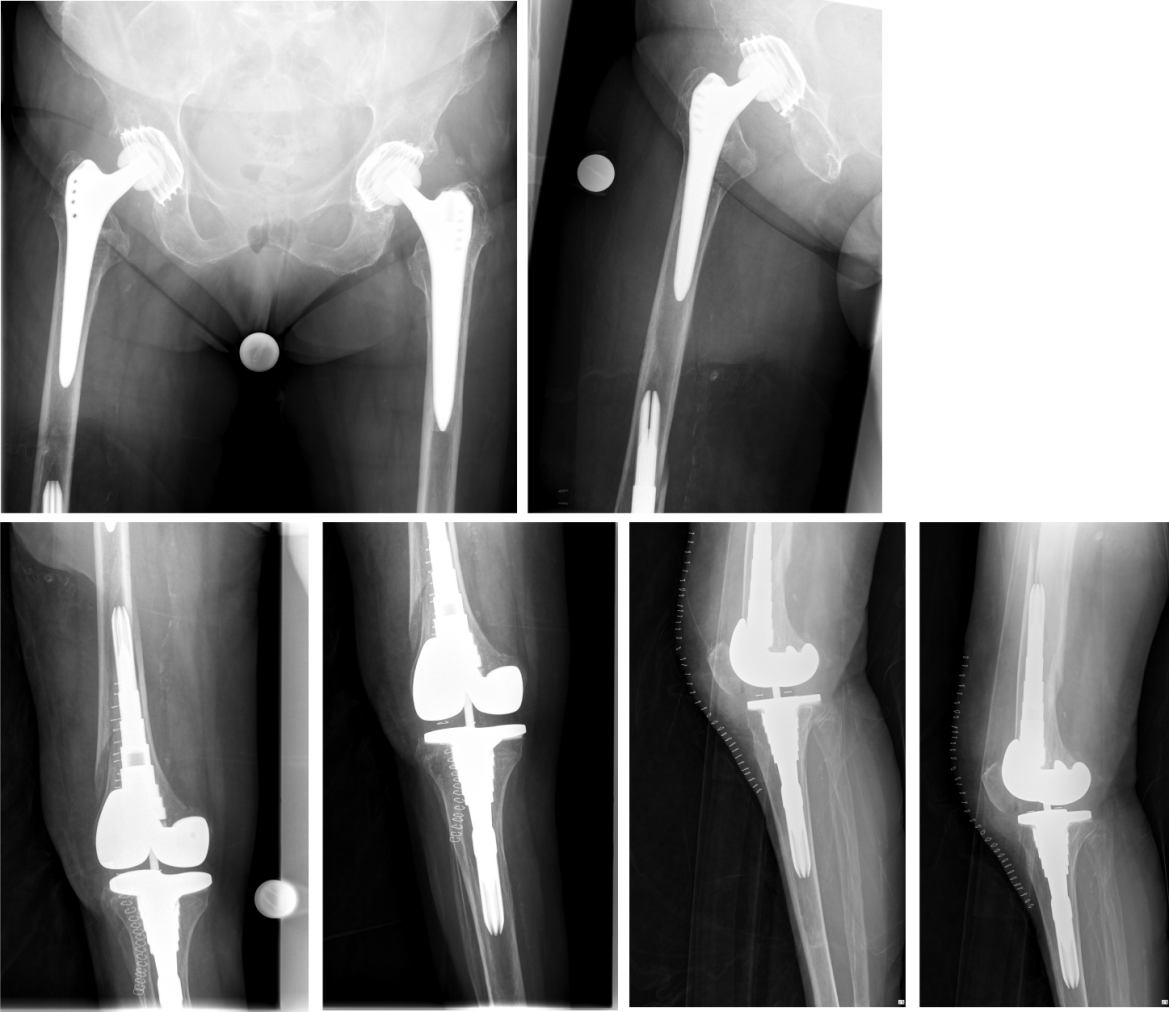
X-ray of the right thigh with the adjacent joints performed immediately after trauma showing the interprosthetic fracture of the femoral shaft

**Figure 4:**
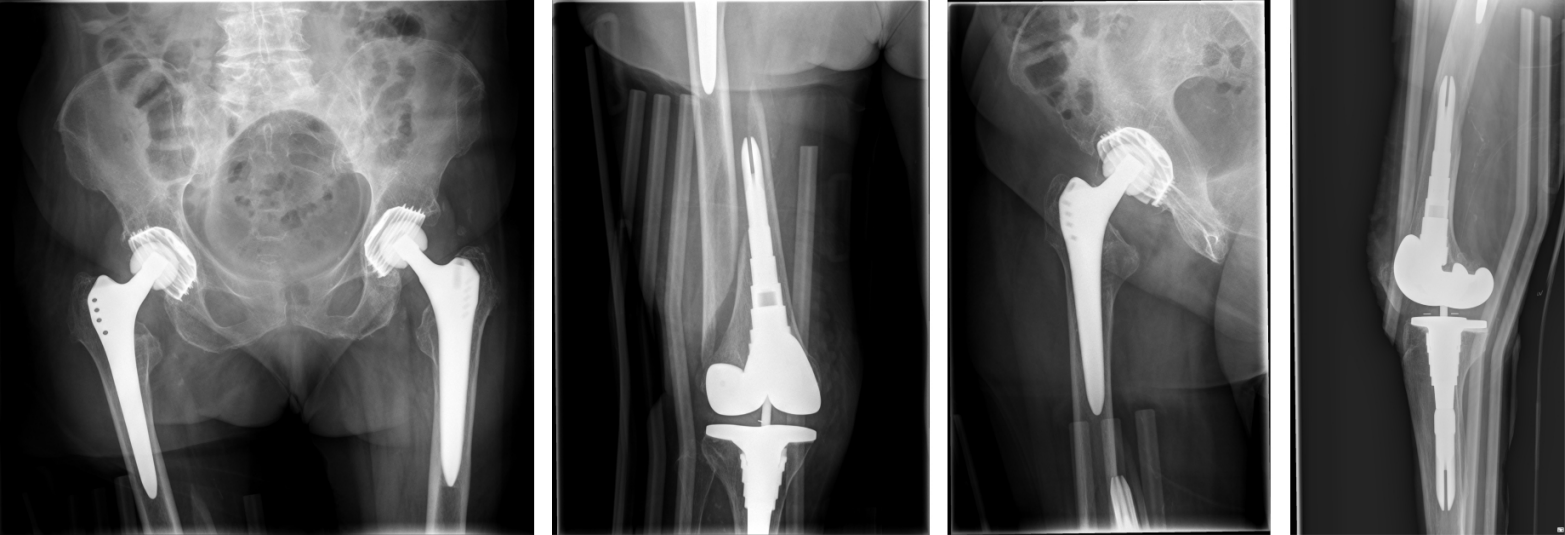
X-ray of the right thigh with the adjacent joints performed prior to surgery using the custom made endoprosthesis

**Figure 5:**
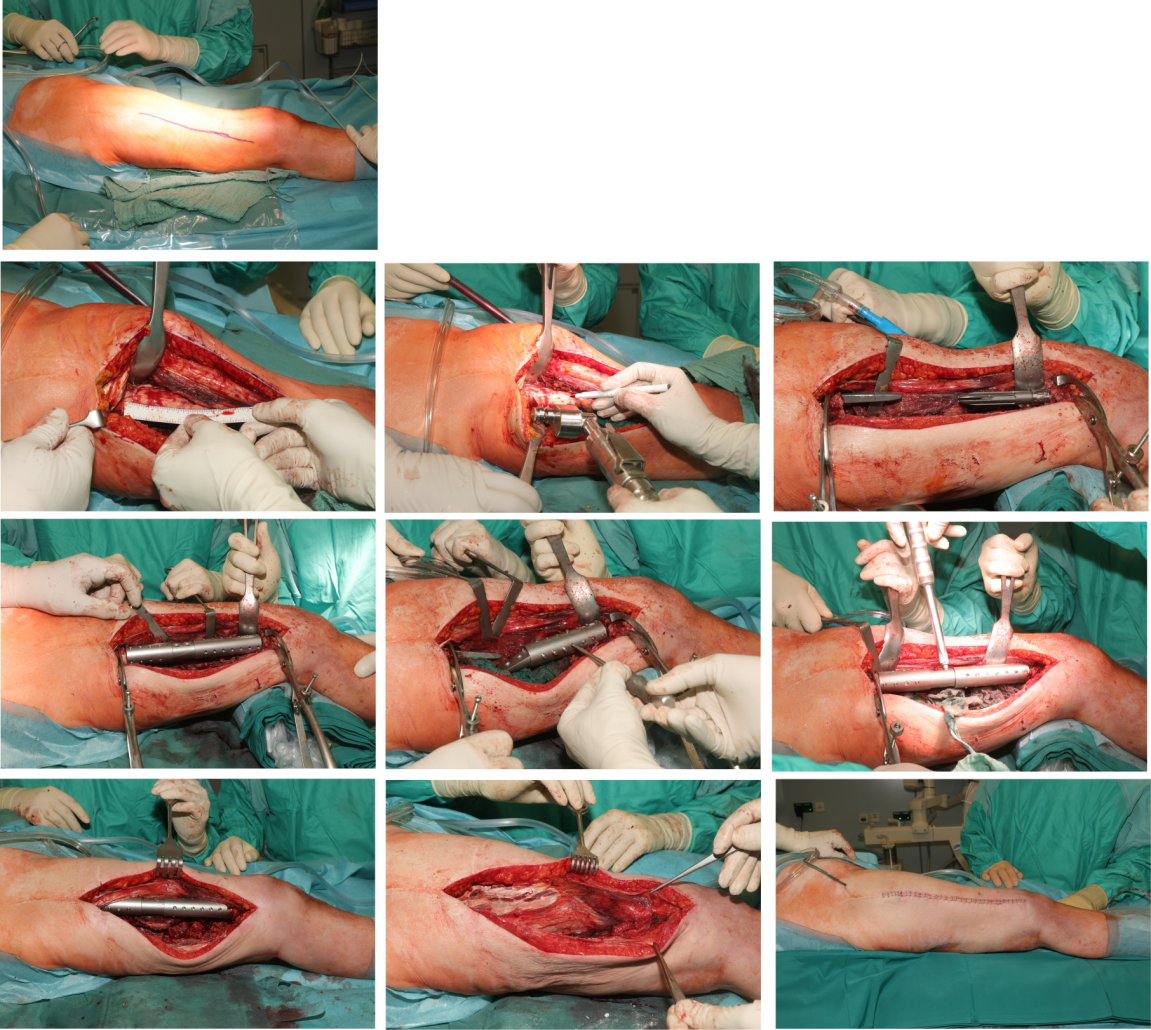
Intraoperative steps Lateral approach to the thigh, exposure of the femoral shaft and the fracture, exact measurements according to preoperative planning to identify the planned level of resection. Exposure of intramedullary femoral stems, the diaphyseal femur the implants were cemented and additionally secured circularly with screws to the two stem parts after setting the correct rotation. Then, the two parts were connected using the screws provided.

Later on, the patient fell during her inpatient stay and suffered from interprosthetic fracture (Figure 3 (Fig. 3), Figure 4 (Fig. 4)). Due to osteoporosis, we favored partial replacement of the diaphysis over open reduction and internal fixation. A custom-made subtotal diaphyseal replacement was planned (Attachment 1) and then delivered within 3 weeks (RescueSleeve^®^, Waldemar Link GmbH & Co. KG, Barkhausenweg 10, 22339 Hamburg, Germany). Despite antithrombotic prophylaxis and mobilization by our physiotherapy, the patient developed an ipsilateral 2-level deep vein thrombosis, which we treated with therapeutic anticoagulation. In preparation for the diaphyseal replacement, a vena cava umbrella was implanted by the angiologists one day prior to surgery. The surgical procedure (Figure 5 (Fig. 5)) was carried out using the lateral approach to the right thigh. After exposure of the femoral shaft and the fracture, exact measurements were carried out according to preoperative planning to identify the planned level of resection (according to the planning 54 mm proximal to the tip of the femoral shaft of the hip and 90 mm distal to the tip of the femoral shaft of the knee). We removed the diaphyseal part of the femur while protecting the soft tissues. Then intramedullary femoral stems were exposed to ensure the correct stem length according to the preoperative planning to ensure sufficient anchoring surface for cementing the clip-on endoprosthetic components. After a previous trial, the diaphyseal femur implants were cemented and additionally secured circularly with screws to the two stem parts after setting the correct rotation. Then, the two parts were connected using the screws provided. After the cement has hardened, the functional test showed very adequate stability, mobility and equal length of both lower extremities. The intraoperative x-ray imaging shows the correct position of the implants. One week after surgery, the vena cava shield was removed without complications. The patient was discharged from hospital ten days after surgery. She was included in the Fracture Liaison Service in order to initiate a bone density measurement (DEXA), an osteological laboratory investigation and the adjustment of antiosteoporotic therapy.

## Results

**Figure 6:**
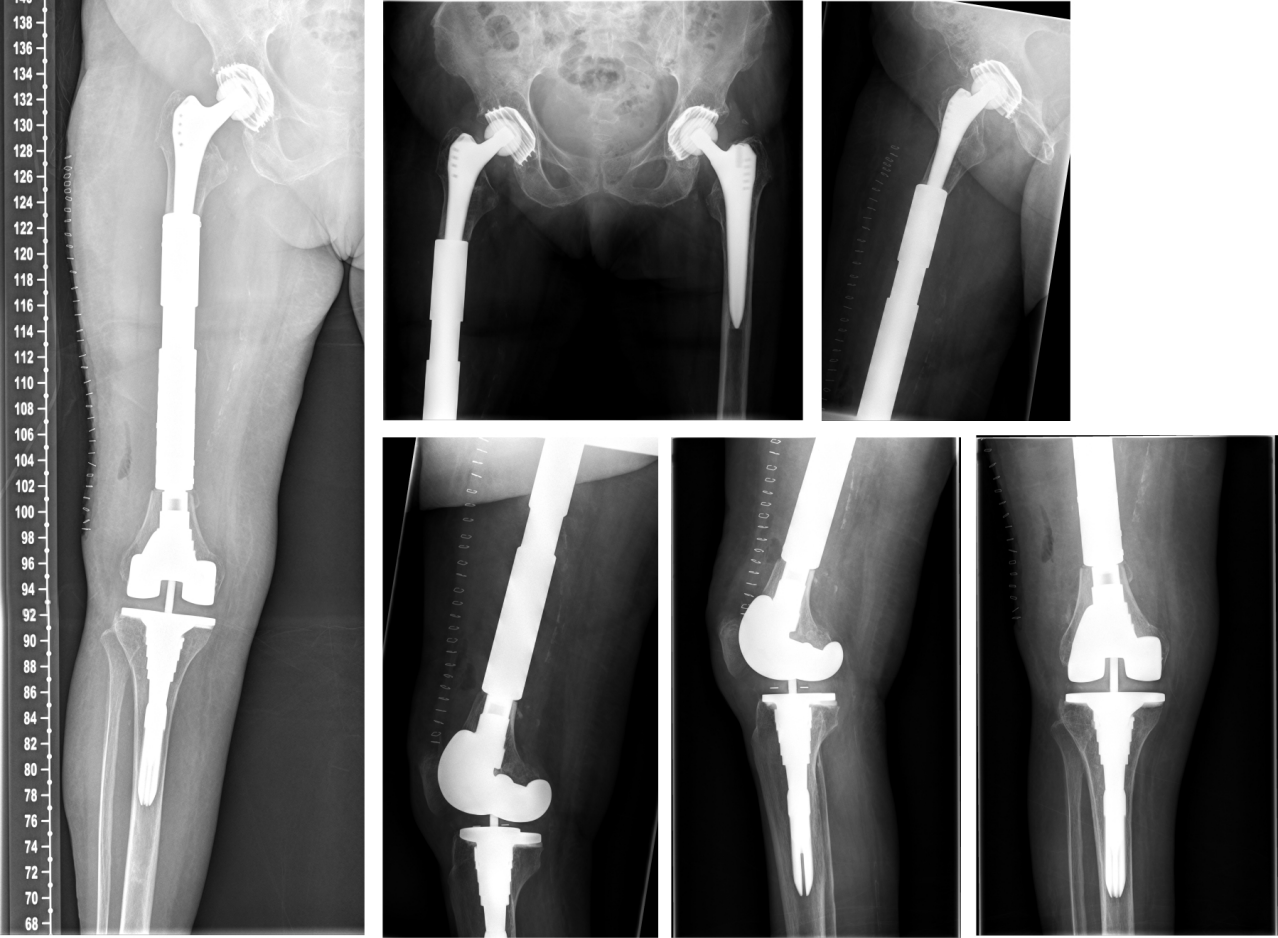
The postoperative radiographs showed correct implant.

The surgical procedure for subtotal replacement of the femoral diaphysis was performed without any intra- or postoperative complication. The duration for the surgical intervention was one hour and 40 minutes. The patient was then mobilized with full weight bearing supervised by physiotherapists at ward level, which she tolerated well. The pain was significantly relieved during hospital stay. The postoperative radiographs showed correct implant position and a satisfactory surgical result (Figure 6 (Fig. 6)).

**Figure 7:**
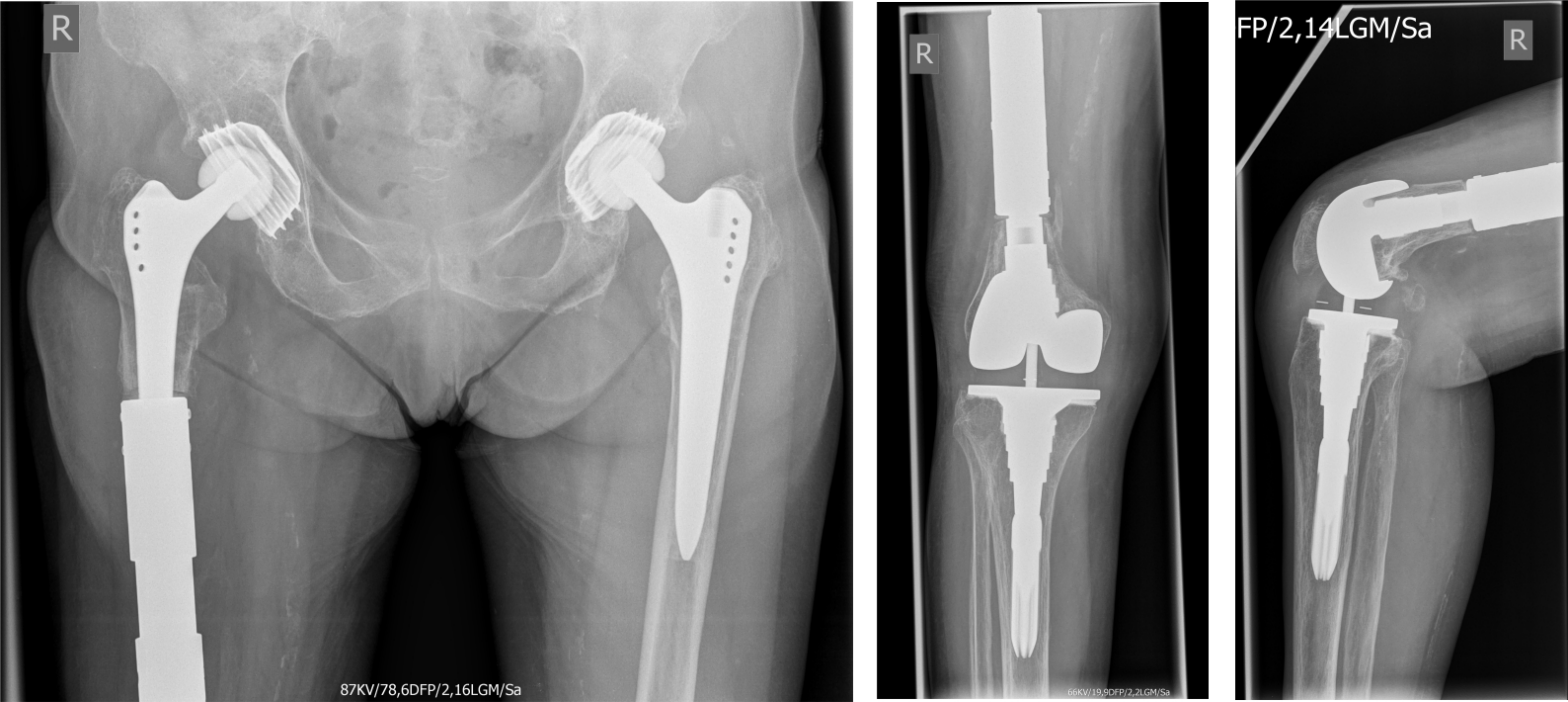
Postoperative radiographs one year after surgery without any morphological changes compared to postoperative radiographs

At one-year follow-up, the patient did not complain of any pain. The Harris Hip Score HHS improved from 26 (prior to partial diaphyseal replacement) to 83 at one-year follow-up (Figure 7 (Fig. 7)), the Western Ontario and McMaster Universities Osteoarthritis Index WOMAC improved from 88 to 16. The range of motion of the right hip joint one year after surgery was: extension/flexion 0/0/90°, abduction/adduction 30/0/20°, external rotation/internal rotation 30/0/20°. The range of motion of the right knee joint one year after surgery was: extension/flexion: 0/0/120°. There were no symptoms or signs of infection or any other complications.

## Discussion

Following the interprosthetic fracture of the diaphysis of the right femur surgery was indicated. After providing the patient with detailed information and presenting the case in the staff meeting, we discussed the following treatment options with the patient:

- Conservative (not medically justifiable because the patient was in pain and had been immobile since the fracture occured)
- Osteosynthesis with a long plate and strut graft (not to be favored as the chance of healing is very limited in the case of significant osteoporosis and insufficient anchoring surface for the osteosynthesis material)
- Change of the femoral component of the knee joint to a long intramedullary shaft and reduction of the fracture and fixation with cerclage. Additionally, additive plate and strut graft at the predetermined breaking point between the two stem ends in the area of the femoral shaft. Due to the significant osteoporosis and the associated limited chance of bone healing, this plan was not favored.
- Cemented implantation of a diaphyseal bone replacement as a bilateral clip-on endoprosthesis that is custom-made. This procedure is off-label, but after planning, it was the safest and most stress-stable treatment option. After consultation with several manufacturers, the average time it takes to manufacture such an endoprosthesis is approximately 6–8 weeks. Due to the supply-chain problems encounterd during the Covid-19 pandemic, the completion time was estimated by most of the implant manufactures to be approximately 3–4 months. However, LINK^®^ was able to produce the above-mentioned implant within 3 weeks. This treatment option was expressly favored in agreement with the patient and therefore implemented.

Mega-endoprostheses are increasingly used in revision arthroplasty of hip and knee joints [1], [2], [3], [4], [5]. The indication for implantation of mega-implants were established in managing major bone defects due to loosening, periprosthetic fractures, re-implantation following periprosthetic joint infection, non-union following fractures as well as complex intraarticular primary fractures [1]. The majority of mega-implant systems provide modular components. The modularity enables a wide range of reconstruction options [1], [2]. However, surgery involving mega-implants is associated with high complication rates [1], [4]. In this particular case, no complications were encountered during or after surgery using the above-mentioned custom-made implant.

The limitation of this study lies in its retrospective design an short follow-up period. However, the patient was 84 years old at the time of surgery and little over 85 at follow-up. Further, most studies on mega-implants encountered in literature are of retrospective design.

## Conclusion

Mega-endoprostheses enable a wide range of management options in the treatment of primary and peri- and interprosthetic fractures of the lower extremities. In many cases, an individual therapeutic plan is necessary ranging up to the use of custom-made implants. 

## Notes

### Ethics approval

Approval of the local institutional review board for study had been given (Ethical Committee at the Medical Faculty, Leipzig University, AZ 020/21-ek) in view of the retrospective nature of the study and all the procedures being performed were part of the routine care.

### Informed consent

The patient has given general consent in the use of their data, including imaging, for analysis and publication. This has been approved by the Ethical Committee. Informed consent was obtained under Ethical approval and consent to participate section.

### Availability of data and material

The datasets used and/or analyzed during the current study are available from the corresponding author on reasonable request.

### Competing interests

The authors declare that they have no competing interests.

### Author’s ORCID

Mohamed Ghanem: 0000-0003-1724-336X

## Supplementary Material

Preoperative planning using the computerized facilities of LINK® (© Waldemar Link GmbH & Co. KG)
