# Supplementary material for: Subtotal diaphyseal replacement of the femur with modular mega-endoprosthesis following interprosthetic fracture. A case report
Source: GMS Interdiscip Plast Reconstr Surg DGPW. 2024 Feb 9;13:Doc01. doi: 10.3205/iprs000183 (PMC10963900; doi:10.3205/iprs000183)
Supplement: Preoperative planning using the computerized facilities of LINK® (© Waldemar Link GmbH & Co. KG) [file IPRS-13-01-s-001.pdf]

Preoperative planning using the computerized facilities of LINK®

(© Waldemar Link GmbH & Co. KG, Helmut D. Link, Barkhausenweg 10, 22339 Hamburg, Germany)

Röntgenbildplanung

Custom-Made:

- Femorale Link Twin RescueSleeve, Tilastan, inkl. Schrauben M5x9, M5x7

Art. Nr.	Artikelbezeichnung	Anzahl	Einheit	Zeit
7712	PE-Einsatz Gr.3/32 zfr.Bicon	1,00		
24041	Titanschale Gr. 3/65 Bicon Porose zfr.	1,00		
1737	Kugelkopf Keramik 32/14 M	1,00		
1889	Schaft Zweymüller Standard Plus Gr.5/14 zfr.	1,00		

Therapie: Implantation einer teilgekoppelten KTEP rechts

Implantate: Fa."DePuy" LCS-VVC
VVC Femurkomponente rechts Gr. Standard zementiert
Femoral Sleeve universal Gr. 31
Universal Stiel 14/75mm
Revisions-Tibia Rotating Plattform M.B.T. Gr.3 zementiert
M.B.T. Revision Metaphyseal Sleeve Gr. 29
Tibia Inlay VVC Gr. 12.5 standard
Universal Stiel 16/75mm

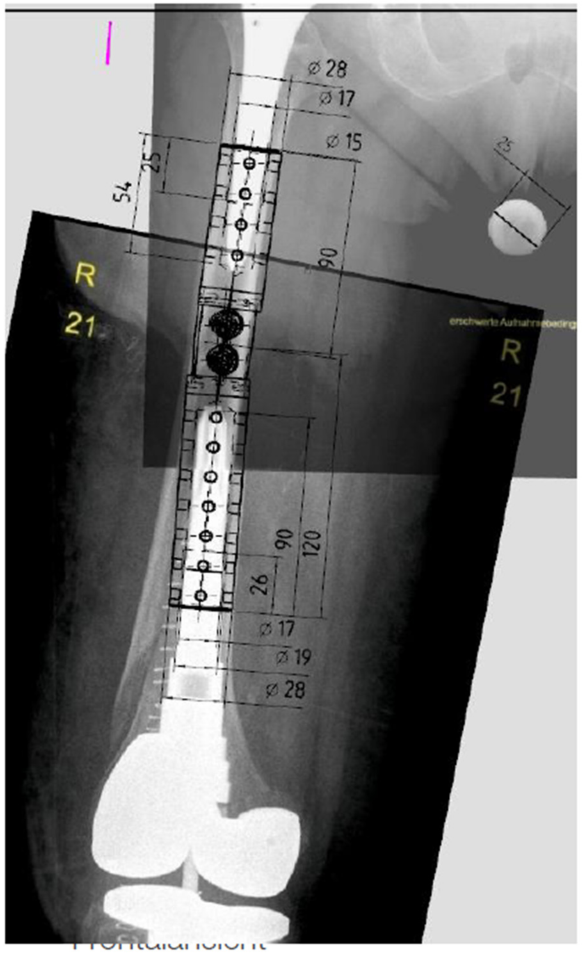

Uns stehen keine Datenblätter mit den Abmaßen der insitu Schäfte zur Verfügung.

© Waldemar Link GmbH & Co. KG – 01.08.2022 – SK – Planung von M. Jakob

Attachment 1 to: Ghanem M, Pempe C, Roth A. Subtotal diaphyseal replacement of the femur with modular mega-endoprosthesis following interprosthetic fracture. A case report. GMS Interdiscip Plast Reconstr Surg DGPW. 2024;13:Doc01. DOI: 10.3205/iprs000183

01

Nach Darstellung des Operationssitus wird die Resektionslänge bestimmt. Anschließend werden die Resektionsstellen am Knochen markiert, wobei die gekoppelte RescueSleeve als Schablone dient.

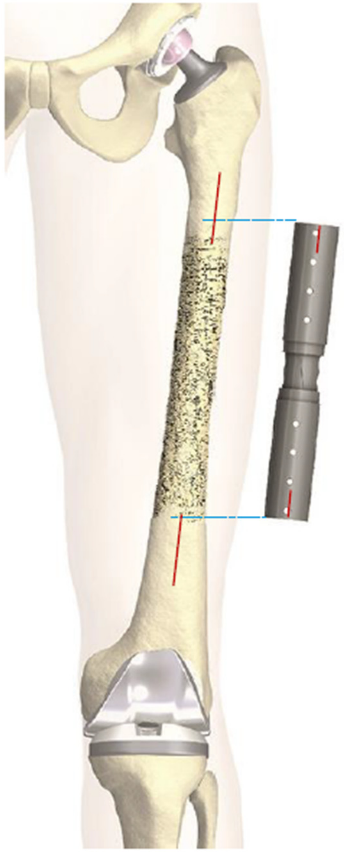

02

Unter Beachtung der Resektionsmarkierungen werden der proximale und distale Schaftanteil der liegenden Prothesen von Knochen befreit. Bei diesem Eingriff müssen temporäre Schutzcerclagen, oder Knochenklemmen verwendet werden.

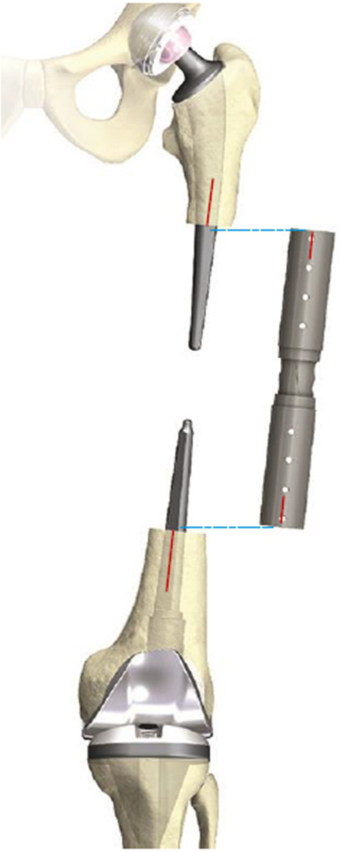

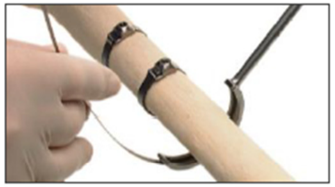

Attachment 1 to: Ghanem M, Pempe C, Roth A. Subtotal diaphyseal replacement of the femur with modular mega-endoprosthesis following interprosthetic fracture. A case report. GMS Interdiscip Plast Reconstr Surg DGPW. 2024;13:Doc01. DOI: 10.3205/iprs000183

03

Zur Probeplatzierung werden zunächst die Schaftfixierschrauben nur so weit in die entsprechenden Öffnungen eingesetzt, dass der Schaft eingeführt werden kann.

Dabei ist zu beachten, dass die Schrauben der proximalen und distalen Hülse aufgrund der unterschiedlichen Schaftdurchmesser am proximalen und distalen Ende unterschiedlich lang sein können - daher dürfen die Schrauben nicht vertauscht und nur für die vorgesehene Hülse verwendet werden.

Außerdem müssen bei beiden Hülsen jeweils zwei Schraubenlöcher in Kupplungsnähe frei bleiben, damit die Luft beim späteren Einbringen des Zements entweichen kann.

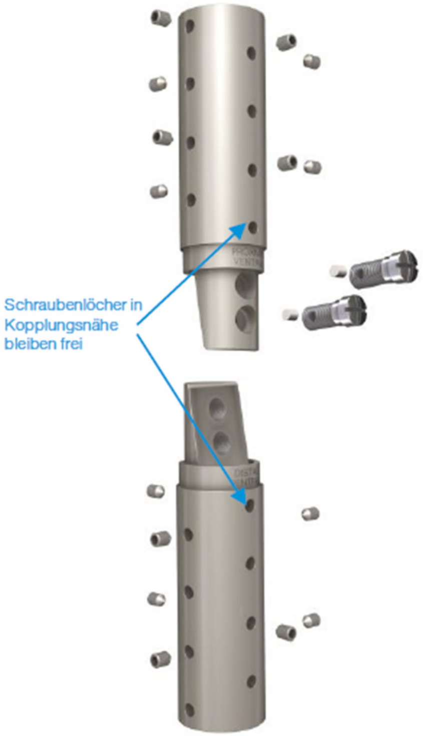

04

Nach provisorischer Fixierung des Schafts an einem Ende, wird der andere Schaft unter Beachtung der Rotationsposition in der anderen Hülse ebenfalls provisorisch fixiert.

Anschließend wird die Rotationsposition mit einem sterilen Stift am Schaft und entsprechend am Rand der Öffnung der zweiten Hülse markiert.

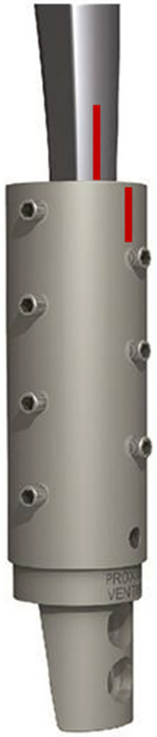

Attachment 1 to: Ghanem M, Pempe C, Roth A. Subtotal diaphyseal replacement of the femur with modular mega-endoprosthesis following interprosthetic fracture. A case report. GMS Interdiscip Plast Reconstr Surg DGPW. 2024;13:Doc01. DOI: 10.3205/iprs000183

09

Anschließend wird der zweite Schaft in die zweite zementgefüllte Hülse eingeführt. Dabei sind die Rotationsmarkierungen zu beachten. Die Schrauben werden vor dem Aushärten des Zements abwechselnd festgezogen. Nach dem Aushärten des Zements werden beide Hülsen in der Taschenkopplung zusammengeführt und mit den beiden Sicherungsschrauben gesichert. Im Bereich des Schraubengewindes befinden sich die Kunststoffstopper.

Hinweis: Die Fixierschrauben haben einen 2,5-mm-Kopf mit Innensechskant.

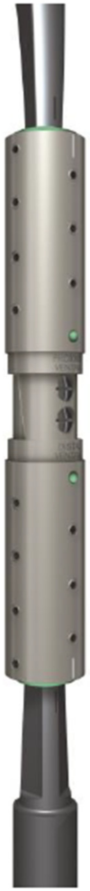

Attachment 1 to: Ghanem M, Pempe C, Roth A. Subtotal diaphyseal replacement of the femur with modular mega-endoprosthesis following interprosthetic fracture. A case report. GMS Interdiscip Plast Reconstr Surg DGPW. 2024;13:Doc01. DOI: 10.3205/iprs000183
